# Decreased swallowing function in the sarcopenic elderly without clinical dysphagia: a cross-sectional study

**DOI:** 10.1186/s12877-020-01832-0

**Published:** 2020-10-21

**Authors:** Yen-Chih Chen, Pei-Yun Chen, Yu-Chen Wang, Tyng-Guey Wang, Der-Sheng Han

**Affiliations:** 1 Department of Physical Medicine and Rehabilitation, National Taiwan University Hospital, No.1, Changde Street, Zhongzheng District, Taipei City, 10048 Taiwan, Republic of China; 2 Physical Medicine & Rehabilitation, National Taiwan University Hospital, Bei-Hu Branch, No.87, Neijiang Street, Wanhua District, Taipei City, Taiwan

**Keywords:** Swallow, Sarcopenia, Hyoid, Tongue, Dysphagia

## Abstract

**Background:**

Sarcopenia and dysphagia are prevalent health issues as the elderly population continues to grow. However, whether sarcopenia, defined by either reduced handgrip strength or gait speed, would lead to pathological effects on swallowing function is still a matter of debate. Studies focusing on subclinical changes in the swallowing function in the sarcopenic elderly are lacking. This study evaluates the swallowing function in the sarcopenic elderly without dysphagia.

Methods: A cross-sectional study was conducted including subjects recruited from the community. Ninety-four individuals aged 65 and older without dysphagia were divided into two groups: sarcopenia and nonsarcopenia. The swallowing assessment included tongue pressure measurement, hyoid displacement (HD), hyoid velocity (HV) measurement with submental ultrasonography, 100-ml water-swallowing test, and the 10-item Eating Assessment Tool (EAT-10).

**Results:**

The average tongue pressure was 47.0 ± 13.7 and 48.6 ± 11.5 kPa in the sarcopenia and nonsarcopenia groups, respectively (*p* = 0.55), whereas the average HD during swallowing was 15.3 ± 4.4 and 13.0 ± 4.2 mm in the sarcopenia and nonsarcopenia groups, respectively (*p* < 0.05). The median of HV during swallowing was 19.5 (6.41–45.86) and 15.9 (3.7–39.7) mm/s in the sarcopenia and nonsarcopenia group (*p* < 0.05). The median of time needed for consuming 100 ml water was 12.43 (3.56–49.34) and 5.66 (2.07–19.13) seconds in the sarcopenia and nonsarcopenia groups, respectively (*p* < 0.05). The median of the EAT-10 score was 0 (0–2) and 0 (0–1) in the sarcopenia and nonsarcopenia groups, respectively (*p* < 0.05).

**Conclusions:**

In elderly individuals, swallowing function was significantly impaired with sarcopenia before clinical symptoms become clear. However, tongue muscles exhibited resistance to sarcopenia. We observed compensative strategies in patients with sarcopenia, such as reduced swallowing speed and increased hyoid bone movement.

## Background

Skeletal muscle mass decreases with advancing age, resulting in decreased strength and functionality. In 1989, Irwin Rosenberg first coined the term “sarcopenia” to describe this phenomenon [1]. Numerous research groups exist worldwide with varying definitions and diagnostic criteria for sarcopenia; however, it is generally agreed upon that sarcopenia has to be defined using a combined approach of muscle mass and muscle quality [2, 3]. According to the Asian Working Group for Sarcopenia (AWGS) diagnostic criteria, sarcopenia should be described as low muscle mass plus low muscle strength (reduced handgrip strength) or low physical performance (reduced gait speed) [3].

Although the swallowing muscles are striated, their embryological characteristics are histologically different from those of the skeletal muscles of the trunk and extremities [4]. A previous study reported that swallowing muscles receive continuous input from the brainstem respiratory center and their activities are synchronized with the rhythmic contractions of the diaphragm [5]. Thus, sarcopenia, defined by the reduced handgrip strength and gait speed [3], does not necessarily lead to pathological effects on swallowing function.

The prevalence of sarcopenic dysphagia is still undetermined [4]. Recently, a growing number of studies demonstrated that sarcopenia might reduce not only body strength but also the strength of swallowing muscles, leading to swallowing function deterioration [6–10]. Most studies included hospitalized and facility-dwelling patients [6–9]; therefore, further studies are warranted involving other settings, such as the community setting. To the best of our knowledge, no studies targeted the swallowing assessment of sarcopenic patients without dysphagia. Discovering the characteristics indicative of the decline in swallowing function is of the utmost importance for providing the appropriate treatment before dysphagia becomes worse.

## Methods

We used tongue pressure measurement, submental ultrasonography, 100-ml water-swallowing test (WST), and the 10-item Eating Assessment Tool (EAT-10) to evaluate the swallowing function in elderly sarcopenic patients without dysphagia. A cross-sectional study was conducted including 94 community-dwelling older individuals (ages above 65). An open announcement was made in Wanhua District, Taipei City, Taiwan, calling for joining the study. The participants were living independently and fully cooperative and were able to eat orally at the time of referral. Exclusion criteria were as follows: People who scored three points or higher on the EAT-10 [11]; those with a history of a neurological disorder such as cerebrovascular diseases, Parkinson’s disease (PD), motor neuron disease, multiple sclerosis (MS), myopathy, and head and neck cancers. According to the AWGS definition, participants were assigned to two groups: sarcopenia or nonsarcopenia (47 individuals each) [3]. We utilized this definition because AWGS collected the best available evidence in sarcopenia research from eastern Asian countries and it was in line with previous reports describing sarcopenia as low muscle mass plus low muscle strength and/or low physical performance. The cutoff values for muscle mass measurements were 7.0 kg/m^2^ and 5.4 kg/m^2^ for men and women using dual X-ray absorptiometry and 7.0 kg/m^2^ and 5.7 kg/m^2^ for men and women using bioimpedance analysis, respectively, whereas the cutoff values for handgrip strength were < 26 kg and < 18 kg for men and women, respectively. The cutoff value for usual gait speed was < 0.8 m/s and the gait speed was casual (at one’s own pace).

Tongue muscle pressure has frequently been used as a measure of swallowing muscle strength [9, 12–15]. Iowa Oral Performance Instrument (IOPI) is one of the most widely used measurement techniques available for assessing the maximum pressure created by contact between the tongue and palate, objectively measuring tongue strength and endurance in practice [13, 16], with good inter- and intrarater reliability [15, 17]. It is a handheld portable device using an air-filled pliable plastic tongue bulb connected with a clear plastic tube, placed on the middle part of the tongue, to estimate the peak pressure exerted on the tongue bulb.

Hyoid movement is essential for the adequate opening of the upper esophageal sphincter (UES) and measured by submental ultrasonography [18, 19]. The protocol was applied and described in detail in previous works [18, 20] as follows. Each participant swallowed 3 mL of water three times where the hyoid bone movement was recorded during swallowing. Then, the best-recorded images of the three attempts were analyzed frame by frame. Moreover, the time interval from the swallow-related hyoid motion onset to the first moment of maximum displacement in the forward movement trajectory was measured. We calculated the speed of hyoid movement as the maximal hyoid bone displacement divided by the time interval.

A 100 ml WST is a sensitive predictor for identifying high-risk patients for swallowing dysfunction. Its sensitivity and specificity were 85.5% and was 50%, respectively [21]. For the WST, we asked participants to consume 100 ml of water in the shortest amount of time. Participants who choked during the test were asked to stop drinking immediately even if they had not finished the water; the protocol was detailed in previous works [21]. Furthermore, we calculated the swallowing speed, as the amount of consumed water divided by the elapsed time.

The EAT-10 is a self-administered, symptom-specific outcome assessment for swallowing dysfunction, demonstrating good consistency, reproducibility, and validity [11]. It is comprised of 10 questions using a five-point Likert scale (0: no problem to 4: severe problem). The total score is ranging from 0 to 40, with the higher scores indicating severe dysphagic symptoms.

We set the effect size as medium, the power as 0.8, and the margin of error as 5% for sample size determination. Additionally, to calculate the sample size, we used G*Power software 3.1 [22]. Therefore, the number of participants required was 94 to an effect size of 0.52. Statistical tests were conducted using IBM SPSS software (SPSS Statistics 20.0; SPSS Inc., Chicago, IL, USA). To assess the normality of data, we employed the Shapiro–Wilk test. The data of sarcopenia and nonsarcopenia groups were compared using a two-sample *t-*test if the values were normally distributed, or the Mann–Whitney *U* test, otherwise. Based on the results of the normality test, the two-sample *t*-test was employed to detect the differences between the groups regarding the maximal pressure generated by contact between the tongue and palate (MTP) and hyoid displacement (HD) during swallowing, whereas the Mann–Whitney *U* test was used for the differences between groups in terms of hyoid velocity (HV) during swallowing, the EAT-10 score, and the swallowing time obtained in the 100 ml WST. The level of significance was 0.05.

## Results

Table 1 list the participants’ characteristics. In the 94 subjects, the mean age was 75.1 ± 5.8 years and 26 (27.7%) were men. Forty-seven of the ninety-four participants were in the sarcopenia group. No significant difference in age or sex was observed between the two groups.

**Table 1:** Characteristics of the study population

	Sarcopenia group (*n* = 47) Mean ± SD	Nonsarcopenia group (*n* = 47) Mean ± SD
Gender		
Male (%)	13 (27.7%)	13 (27.7%)
Female (%)	34 (72.3%)	34 (72.3%)
Age (years)	75.2 ± 6.3	75.1 ± 5.4

A two-sample *t*-test was used to identify the differences between groups

*SD* standard deviation

Table 2 summarizes the swallowing factors in the sarcopenia and nonsarcopenia groups. According to the results of the normality test, MTP and HD complied with normal distribution, represented by mean followed by the standard deviation. However, EAT-10, WST, and HV did not comply with the normal distribution, represented by median followed by range. The EAT-10 score was significantly lower in the nonsarcopenia group than that of the sarcopenia group. The swallowing time for 100 ml WST was significantly longer and the HD and HV during swallowing were significantly greater in the sarcopenia group than those in the nonsarcopenia group. No significant difference was observed regarding maximal tongue pressure between the two groups.

**Table 2:** Sarcopenia group and nonsarcopenia group outcomes

	Sarcopenia group (*n* = 47)	Nonsarcopenia group (*n* = 47)
MTP (kPa)	47.0 ± 13.7	48.6 ± 11.5
HD (mm)	15.3 ± 4.4	13.0 ± 4.2*
HV (mm/s)	19.5 (6.41–45.86)	15.9 (3.7–39.7)*
EAT-10	0 (0–2)	0 (0–1)*
WST (second)	12.43 (3.56–49.34)	5.66 (2.07–19.13)*

*MTP* maximal pressure generated by contact between the tongue and palate, *HD* hyoid displacement during swallowing, *HV* hyoid velocity during swallowing, *EAT-10* the 10-item Eating Assessment Tool score, *WST* swallowing time obtained in the 100-ml water-swallowing test, *p < 0.05

A two-sample *t*-test was used to identify the differences between groups in MTP and HD, while the Mann–Whitney *U* test was used for HV, EAT-10, and WST

## Discussion

In this study, we assessed the swallowing function in elderly sarcopenic patients without dysphagia. The EAT-10 score was significantly greater in the sarcopenia group than that in the nonsarcopenia group; however, despite the statistical difference, none of the participants’ scores reached the cut point of 3, suggesting dysphagia. Therefore, the differences in EAT-10 scores between the two groups should be interpreted with caution.

The time needed to consume 100 ml of water was significantly longer in the sarcopenia group than that in the nonsarcopenia group, suggesting the swallowing function is reduced in patients with sarcopenia. Buchholz et al. postulated that in dysphagic patients, the swallowed bolus size might be reduced as a compensation strategy, resulting in a slower swallowing speed [23]. Nathadwarawala et al. discovered that, in subjects with swallowing problems, objective swallowing speed was significantly diminished [24]. Swallowing speed less than 10 ml/s is considered to be a strong predictor of dysphagia [25]. Although our sarcopenic participants have not developed dysphagia, the delay in completing the swallowing test reflects the compensatory mechanism (decreased volume per swallow) they developed before clinical problems become apparent [23, 24]. To the best of our knowledge, this is the first study to 100 ml WST on elderly individuals with sarcopenia. Yoshitoshi et al. evaluated the swallowing function in bedridden older adults using various bolus sizes (2, 3, and 5 ml of liquid), where the swallowing performance was significantly correlated with the mid-upper arm circumference, rather than the general frailty [26]. The overall reduction of lean body mass might involve not only the mid-upper arm circumference but also the swallowing muscles.

The sarcopenia group had greater HD when swallowing 3 mL of water than the nonsarcopenia group. According to our knowledge, this is the first study investigating the relationship between hyoid bone movement and sarcopenia. There was only one case report about a patient with sarcopenic dysphagia, reporting that both the maximal amounts of HD and HV during swallowing were increased after rehabilitation [27]. Previous research revealed that in an older population with dysphagia, HD was greater than the normal level in small bolus ingestion; however, the magnitude of HD decreased to normal or was below normal levels in large bolus ingestion [28]. It was hypothesized that greater displacement indicated compensation for insufficient UES opening, which could no longer be maintained with larger boluses in dysphagic patients [28]. Therefore, our findings included the clinical application of restricted bolus volume in sarcopenic patients with or without dysphagia. Meanwhile, rehabilitative programs for strengthening the hyoid elevation muscles might be useful [27].

Moreover, the sarcopenia group had greater HV when swallowing 3 mL of water than that in the nonsarcopenia group, which involves HD and duration. Previous studies hypothesized that HV is more sensitive in detecting changes in swallowing kinematics than HD alone [29] since studies of age-related changes in HD obtained heterogeneous results [30–32]. Furthermore, the decreased HV may slow down the closure of the laryngeal vestibule and opening of UES, thus increasing the risk of aspiration [33]. Many studies have already incorporated HV to describe hyoid excursion kinematics during swallowing [29, 33]. For instance, Ueda et al. [34] reported decreased hyoid excursion velocity in dysphagic patients; alternatively, previous studies showed that older healthy subjects had greater HV during swallowing than their younger counterparts [29, 35], supporting the concept of adaptation and compensation in the healthy older individuals. Greater HV in our sarcopenic participants may indicate a compensatory response to subclinical changes in the swallowing mechanism. These interpretations are speculative and require future experimental evaluation; however, the results of this study suggest that HV may be a sensitive indicator when assessing the effects of sarcopenia on swallowing performance.

Our results revealed that there was no significant difference between the sarcopenia group and their healthy counterparts regarding tongue pressure. Studies have reported that tongue strengthening could enhance the maximum peak lingual pressures in both healthy adults and dysphagic patients; however, whether the gain in lingual pressures generalizes to the functional improvement in swallowing is still undetermined [36, 37]. A previous study done in Japan demonstrated that reduced tongue pressure is correlated with older individuals with sarcopenia [9]; however, 42.3% of their subjects suffered from dysphagia, and all their subjects were hospitalized at the time of enrollment. Besides, they only included older subjects who were at least 75 years old. This study only enrolled community-dwelling elderly individuals without dysphagia. Another explanation was based on histological evidence as follows. The swallowing muscles and somatic muscles stem from different embryological origins and the swallowing muscles were continuously activated by the brainstem respiratory center [5]. Many common sarcopenic muscle characteristics are rare in the lingual muscles of rats, such as type II muscle fiber atrophy, and change to slower myosin heavy chain isoforms and neuromuscular junction dysmorphology [38, 39]. In particular, the styloglossus muscle was unaffected by sarcopenia anatomically and molecularly [40]. Besides, it was reported that the elderly can still produce similar noneffortful and effortful swallow pressures compared to younger adults [14] and tongue functional reserve does not decline significantly with age [41]. These findings suggest that tongue muscles are not as susceptible to sarcopenia as appendicular muscles and lingual function does not necessarily deteriorate with age.

This study had several limitations. First, we recruited our participants in annual health exams occurring in a local community hospital. Younger elderly individuals were more likely to receive recruiting information and join our study, whereas older people were less likely to be involved. Second, regarding WST, we did not measure the number of sips. Third, we did not assess the muscle mass related to swallowing. Fourth, we could not stratify the patients with sarcopenia according to the severity of the disease. Fifth, this study was conducted in a single region and included only community-dwelling elderly individuals without dysphagia within that location. A follow-up study including an expanded target area and sarcopenic patients with dysphagia is warranted.

## Conclusions

Swallowing function is significantly reduced in elderly individuals with sarcopenia, even before the clinical symptoms become clear. However, tongue muscles appear to be resistant to sarcopenia. We observed compensative strategies in our sarcopenic participants, such as reduced swallowing speed and increased hyoid bone movement.

## Data Availability

The datasets used and/or analyzed during the current study are available from the corresponding author on reasonable request.

## Abbreviations

AWGS: Asian Working Group for Sarcopenia
EAT-10: 10-item Eating Assessment Tool
MS: Multiple sclerosis
PD: Parkinson’s disease
UES: Upper esophageal sphincter
WST: 100 ml water-swallowing test
